# A hypothyroid mother after subtotal thyroidectomy delivered a newborn with hyperthyroidism from fetal stage: a case report

**DOI:** 10.1186/s12884-022-04654-7

**Published:** 2022-04-12

**Authors:** Cheng Peng, Weijie Sun, Lixin Fan, Li Li, Xiaojiao Zhang, Ying Gao, Xinlin Hou

**Affiliations:** 1 Department of Pediatrics, Peking University First Hospital, Beijing, 100034 China; 2 Department of Obstetrics and Gynecology, Peking University First Hospital, Beijing, 100034 China; 3 Department of Endocrinology, Peking University First Hospital, Beijing, 100034 China

**Keywords:** Neonatal hyperthyroidism, Maternal subtotal thyroidectomy, Thyroid function, Thyroid-stimulating hormone receptor antibody, Antithyroid drug

## Abstract

**Background:**

Neonatal hyperthyroidism is an extension of fetal disease. Most cases of neonatal hyperthyroidism are transient but may excessively harm multiple organ functions through the actions of maternal thyroid-stimulating hormone receptor antibodies on the neonatal thyroid gland.

**Case presentation:**

The hyperthyroid mother underwent subtotal thyroidectomy before pregnancy and regularly took levothyroxine to avoid hypothyroidism, but still had a high-level thyroid-stimulating hormone receptor antibody (TRAb). The neonate suffered from hyperthyroidism due to the transplacental TRAb. After a regular medication schedule of an antithyroid drug, combined with a β-blocker to control the ventricular rate, the infant gradually recovered, allowing normal motor and intellectual development.

**Conclusions:**

Maternal subtotal thyroidectomy cannot prevent the secretion of thyroid receptor antibodies, which may cause either hypothyroidism or hyperthyroidism. The balance between antithyroid drugs and levothyroxine is critical in clinical practice.

## Background

Fetal hyperthyroidism usually occurs in the second and third trimesters of pregnancy. Neonatal hyperthyroidism tends to be an extension of disease sustained in utero; it is harmful in the fetal and neonatal periods and requires guideline-directed clinical management. Most neonatal hyperthyroidism is transient, commonly caused by stimulation by maternal thyroid-stimulating hormone receptor antibody (TRAb) crossing the placenta, resulting in a baby’s high thyroid hormone secretion. In rare situations, thyroid disease is associated with a genetic variation in the thyroid-stimulating hormone receptor (TSHR) or G-protein alpha stimulatory subunit (GNAS) gene such as in McCune-Albright syndrome [1].

The prevalence of neonatal hyperthyroidism is 1 in 25,000 newborns to 1 in 50,000 newborns [1]. The most severe hazard in utero is prolonged tachycardia, leading to increased cardiac output, cardiac failure, and even intrauterine fetal demise. The clinical manifestations in the neonatal period are tachycardia, irritability, hyperphagia without weight gain, goiter, thyroid eye disease, small anterior fontanel, advanced bone age, hepatomegaly, and/or splenomegaly. Severely affected patients could present with cardiac failure, craniosynostosis, microcephaly, psychomotor disabilities, and even death [2]. TRAb levels should be measured in cord blood at delivery; nearly one-third of infants with increased cord blood TRAb levels develop neonatal hyperthyroidism. A newborn with such symptoms should receive timely medical intervention. If timely diagnosis and proper treatment are obtained, the prognosis of most patients is favorable. Consequently, clinicians need to monitor maternal and fetal thyroid function.

In this study, we revisit a case of a newborn child with a perinatal history of maternal thyroid disorder.

## Case presentation

The 31-year-old mother had hyperthyroidism five years prior and took oral methimazole accordingly; however, the mother’s TRAb remained stable at over 40 IU/ml, refractory to treatment. She had undergone a spontaneous abortion three years prior and consequently underwent subtotal thyroidectomy one year prior to control the thyroid function. She regularly took levothyroxine sodium (LT4) postoperatively, and the patient was assessed to have normal thyroid function with elevating TRAb concentration from pre-gestation (T_3_ 1.99 nmol/L, fT_3_ 5.10 pmol/L, T_4_ 145.5 nmol/L, fT_4_ 17.83 pmol/L, TSH 0.58 μIU/ml, TRAb > 40 IU/ml) to the second trimesters of pregnancy (> 40 IU/ml). However, at 32 weeks of gestation, the fetal heart rate increased to 172 beats per minute, persistent for over 10 min during the electronic fetal monitoring, and thyroid ultrasound revealed fetal goiter with decreased echo. The mother The dimensions of the fetal thyroid were 75.2 mm. The obstetrician suggested withdrawing levothyroxine and introducing methimazole at a dose of 15 mg per day. In the latter part of pregnancy, we arranged pregnancy check-ups weekly. Close fetal heart rate monitoring fluctuated between 140 and 160 beats per minute. The fetal ultrasound showed neither hydrops fetalis nor cardiac hemodynamic changes. The multi-disciplinary team proposed initiating a therapeutic trial that involved concurrently administering methimazole 15 mg and levothyroxine sodium 125 μg until delivery. We monitored maternal heart rate, complete blood count, and liver function and advised adding metoprolol if necessary. The indicators above are normal, and the patient had no associated symptoms. The details of the laboratory tests of thyroid function are listed in Table 1.

**Table 1:** Thyroid function tests of the mother before and during pregnancy

Gestation weeks	Bodyweight (kg)	T_3_ nmol/L	fT_3_ pmol/L	T_4_ nmol/L	fT_4_ pmol/L	TSH μIU/ml	TRAb IU/ml	Treatment	
								LT4(μg)	Methimazole (mg)
Pre-gestation	53.4	1.99	5.10	145.5	17.83	0.58	> 40	50	/
1^+ 2^	/	2.12	5.26	134.5	19.46	0.75	/	50	/
4^+ 2^	/	1.66	4.56	138.71	12.71	2.40	/	75	/
6^+ 6^	/	2.26	5.27	168	20.53	0.66	/	62.5	/
9^+ 6^	/	/	/	/	17.55	0.14	/	62.5	/
13^+ 1^	/	3.22	5.30	238.2	21.28	0.04	/	50	/
16^+ 6^	/	3.01	4.98	243.5	18.04	0.04	/	25	/
20^+ 2^	58.8	3.11	4.01	162.5	13.92	3.04	39.46	25 × 4d + 50 × d	/
23^+ 2^	/	2.54	3.70	138	13.06	4.39	/	50 × 5d + 75 × 2d	/
26^+ 2^	65	3.56	4.79	193	15.96	0.49	/	50	/
30^+ 2^	66.2	3.58	5.03	232.9	17.04	0.13	> 40	25	/
32^+ 2^ **(fetus goiter)**	67.3	3.03	4.89	205.2	16.35	0.2	/	Withdrawal	/
33	/	/	/	/	/	/	/	/	15
34^+ 1^	68.4	2.31	3.65	144.1	13.91	1.82	/	50	10/5 alternating
35^+ 1^	68.9	2.27	3.52	128.3	10.08	6.35	/	75	10/5 alternating
36	70.5	2.49	3.85	146.7	11.76	15.02	86.3	125	15
37	69.8	2.71	4.31	170.9	15.57	4.16	/	125	15
38	70	2.64	4.31	232.4	16.69	1.08	/	125	15

*T*_*3*_ triiodothyronine, *fT*_*3*_ free triiodothyronine, *T*_*4*_ thyroxine, *fT*_*4*_ free thyroxine, *TSH* thyroid-stimulating hormone, *TRAb* thyroid-stimulating hormone receptor antibody, *LT4* levothyroxine sodium

A male neonate, appropriate for gestational age, was born by vaginal delivery at 37 weeks and five days with a weight of 3130 g. The newborn had no fetal distress, no maternal history of premature rupture of membranes, or neonatal asphyxia. At birth, the placenta and appendages were normal. The primary reason for hospitalization was a thyroid disorder since the gestational age of 32 weeks. Upon delivery, the child’s physical examination revealed stable vital signs, irritability, small anterior fontanel, proptosis, enlarged cardiac borders on percussion, and normal heart rate for age. The main objective of hospitalization was evaluating the primary disease and its complications.

We actively carried out relevant laboratory tests and targeted treatments for thyroid disorders. The umbilical arterial thyroid function test showed triiodothyronine (T_3_) 1.36 nmol/L (0.49–3.30 nmol/L), free triiodothyronine (fT_3_) 3.15 pmol/L (3.50–6.50 pmol/L), thyroxine (T_4_) 145.5 nmol/L (151.9–198.6 nmol/L), free thyroxine (fT_4_) 19.04 pmol/L (11.48–22.70 pmol/L), thyroid-stimulating hormone (TSH) 0.01 μIU/ml (1.10–19.12 μIU/ml). The serum anti-thyroglobulin antibodies (TgAb; 16.71 IU/ml) were noted, and TRAb exceeded 40 IU/ml. The thyroid ultrasound revealed an enlarged thyroid gland, and the width of the left lobe, right lobe, and isthmus of the thyroid gland was 1.1 cm, 1.2 cm, and 0.3 cm, respectively. The initial chest radiograph suggested cardiac enlargement; however, myocardial enzymes and B-type natriuretic peptide were within normal ranges. The ultrasonic cardiogram on day two showed patent ductus arteriosus (5.1 mm × 2.1 mm), and an average left ventricular ejection fraction (74%). Holter monitoring showed a heart rate of 140–200 beats per minute for 36% of the day. On the third day of life, the patient was started on a 0.4 mg of oral methimazole regimen every 12 h, at 0.25 mg/kg/day. On day seven, the patient was administered propranolol 0.5 mg every 8 h. We subsequently achieved reasonable control of the heart rate. During hospitalization, we regularly monitored thyroid function tests and TRAb levels. There was a gradual downward trend on T_4_, while the T_3_, fT_3_, and fT_4_ had no evident changes. The patient’s condition was deemed stable; meanwhile, the substantial value of TRAb was assayed for the first time before discharge. He was subsequently discharged for further outpatient follow-ups. On the 29th day of life, the TRAb level fell to 23.13 IU/ml for the first time in the patient’s clinical course. On the 51st day of life, the gradually diminishing T_4_, fT_4_, and TRAb (10.96 IU/ml) levels combined with recovering TSH levels (0.53μIU/ml) prompted us to halve the dosage of methimazole (0.125 mg/kg). On the 88th day of life, the follow-up studies showed the thyroid function test and TRAb level. Eventually, on day 98, the neonatologist discontinued all medications onboard. Meanwhile, the patient underwent a developmental assessment on routine follow-ups, appropriate for age. Details of thyroid function tests are summarized in Table 2.

**Table 2:** Thyroid function tests over the first 88 days of the infant’s life

Date	T_3_ nmol/L	fT_3_ pmol/L	T_4_ nmol/L	fT_4_ pmol/L	TSH μIU/ml	TRAb IU/ml	Treatment
Day 0	1.36	3.15	145.5	19.04	0.01	> 40	
Day 2	2.46	6.49	183.6	23.46	0.01	> 40	methimazole 0.25 mg/kg/day on Day 3
Day 5	2.5	5.28	188.6	19.77	0.01		↓
Day 11	3.0	7.26	151.8	20.62	0.01	65 (diluted)	↓
Day 29	3.5	8.8	115.9	17.58	0.01	23.13	↓
Day 51	2.07	4.69	73.7	10.18	0.53	10.96	methimazole 0.125 mg/kg/day
Day 58	2.01	4.29	64.4	8.7	2.51	/	↓
Day 88	3.1	8.05	128	19.24	0.11	3.87	withdrawal

*T*_*3*_ triiodothyronine, *fT*_*3*_ free triiodothyronine, *T*_*4*_ thyroxine, *fT*_*4*_ free thyroxine, *TSH* thyroid-stimulating hormone, *TRAb* thyroid-stimulating hormone receptor antibody

## Discussion and conclusion

Previous studies have shown that untreated or even partially treated overt hyperthyroidism during pregnancy could increase the risk of pregnancy loss and fetal mortality [3, 4]. Women diagnosed with hyperthyroidism should counsel the endocrinologist regarding plans for pregnancy and the adverse effects of both antithyroid drugs and LT4 during pregnancy [5]. Some studies showed that treatment with surgery before pregnancy is recommended for those who desire future pregnancy with a high TRAb level [6–8]. If surgery is undertaken, hypothyroidism should be treated with levothyroxine to achieve a euthyroid state before trying to conceive. Next, according to the guideline by American Thyroid Association in 2017, a maternal serum determination of TRAb is recommended at initial thyroid function testing during early pregnancy among all women with a prior history of hyperthyroidism treated with surgery [9, 10].

In this case, the mother underwent a prior miscarriage due to thyroid disorder. She underwent surgery to prevent fetal and neonatal thyroid disorder. However, the stubborn elevating TRAb level at a critical stage of pregnancy resulted in fetal and neonatal hyperthyroidism and tachycardia. Considering the risk of intrauterine growth restriction, fetal bradycardia, and neonatal hypoglycemia, [11] the obstetrics and gynecology team decided against maternal administration of propranolol. It is worth noting that there is a subtle balance between maternal TRAb and thyroid hormones. The complicated regulation of maternal thyroid function is a rigorous challenge for medical intervention. Despite thyroidectomy to avoid miscarriage, the human body still secretes TRAb, which crosses transplacentally to enter the fetal circulation, resulting in hyperthyroidism during the fetal and neonatal periods. Taking ATDs could inhibit the synthesis of T_3_ and T_4_. Meanwhile, levothyroxine could protect against resultant hypothyroidism.

In this therapeutic dilemma of maternal hypothyroidism with secondary fetal hyperthyroidism, medical procedures should focus on (1) regular thyroid function screening during pregnancy. The diagnostic criteria for maternal hyperthyroidism include goiter, tachycardia (> 120 beats per minute), ophthalmopathy, and weight loss. The key laboratory finding is the thyroid function test, primarily T_3_, TSH, and TRAb titers [9]. (2) For women with hyperthyroidism who had previously been treated with radioactive iodine, surgery, or taking oral ATD, serum TRAb was recommended to detect in the first trimester of pregnancy. If serum TRAb is negative early in pregnancy, retesting is not required. If the result presents an elevating TRAb, retesting at 18 to 22 weeks of gestation is recommended. Serum TRAb should be tested again during the third trimester and evaluate the need for fetal and neonatal monitoring on the condition that the repeated measurement is elevated or ATD is initiated [12]. Combining the guideline in China with the clinical situations of our pregnant mother, we designed a meticulous scheme to facilitate clinical decision-making [13]. Once maternal thyroid function disorder is biochemically detected, medications are subsequently titrated to maintain a stable thyroid function for an asymptomatic status. Based on keeping normal thyroid function of the mother, clinicians should pay more attention to reasonable stability of fetal condition, including maintaining normal thyroid appearance, regional blood flow, in utero growth, and fetal heart rate. The fetus should be monitored every 4–6 weeks from mid-pregnancy until birth [14]. According to this case experience, the level of thyroid function is not parallel with the TRAb. The general treatment principle is to maintain a constant maternal thyroid function, reducing fetal complications. The goal is to prepare for the transition to neonatal treatment. (3) Hyperthyroidism patients with good antithyroid drug adherence have a lower risk of severe complications [15]. The adherence of pregnant women to therapy is of primary importance. There is no consensus on the best practice for the dosage of ATD, and the treatments are empirical. According to the condition of pregnant women and the degree of fetal goiter, the multi-disciplinary team should draw up an individual schedule. The lowest effective dose may be applied during the therapy of fetal hyperthyroidism to minimize the risk of fetal hypothyroidism. Maintaining maternal serum fT_4_ levels at or moderately above the upper limit of the reference range is appropriate for euthyroid fetal status.

In summary, thyroid disorders are one of the main topics in pregnancy complications. At present, more attention has been paid to the effects of hormone abnormalities on infertility, pregnancy outcomes, and long-term motor and intellectual development in offspring. Maternal and fetal diseases identification has gradually improved with the development of endocrinology, perinatology, obstetrics and gynecology, fetal medicine, pediatrics, genetics, and ultrasonic medicine. The multi-disciplinary team makes it possible to manage both maternal and fetal/neonatal thyroid disease from the prenatal period to post-delivery. For a mother with subtotal thyroidectomy, the maternal body may be in a state of hypothyroidism and high-level serum TRAb. It is challenging for clinical decisions to decrease the serum level. Identifying and managing fetal/neonatal thyroid diseases at an early stage of life and reducing long-term comorbidities are crucial issues that need to be solved. Therefore, monitoring fetal surveillance and dosage adjustment to relieve the symptomatic fetal effect without deteriorating the mom is critical.

## Data Availability

The datasets used and analyzed in the case report are available from the corresponding author upon reasonable request.

## Abbreviations

TRAb: Thyroid-stimulating hormone receptor antibody
LT4: Levothyroxine sodium
ATD: Antithyroid drug
T_3_: Triiodothyronine
fT_3_: Free triiodothyronine
T_4_: Thyroxine
fT_4_: Free thyroxine
TSH: Thyroid-stimulating hormone
TgAb: Anti-thyroglobulin antibody
TSHR: Thyroid-stimulating hormone receptor
GNAS: G-protein alpha stimulatory subunit
